# Neuroimaging Studies of Antidepressant Placebo Effects: Challenges and Opportunities

**DOI:** 10.3389/fpsyt.2019.00669

**Published:** 2019-09-24

**Authors:** Vanessa Brown, Marta Peciña

**Affiliations:** Department of Psychiatry, University of Pittsburgh, Pittsburgh, PA, United States

**Keywords:** antidepressants, placebo, neuroimaging, computational psychiatry, depression

## Abstract

Over the last two decades, neuroscientists have used antidepressant placebo probes to examine the biological mechanisms implicated in expectancies of mood improvement.However, findings from these studies have yet to elucidate a model-based theory that would explain the mechanisms through which antidepressant expectancies evolve to induce persistent mood changes. Compared to other fields, the development of experimental models of antidepressant placebo effects faces significant challenges, such as the delayed mechanism of action of conventional antidepressants and the complex internal dynamics of mood. Still, recent neuroimaging studies of antidepressant placebo effects have shown remarkable similarities to those observed in other disciplines (e.g., placebo analgesia), such as placebo-induced increased µ-opioid signaling and blood-oxygen-level dependent (BOLD) responses in areas involved in cognitive control, the representation of expected values and reward and emotional processing. This review will summarize these findings and the challenges and opportunities that arise from applying methodologies used in the field of placebo analgesia into the field of antidepressant placebo effects.

## Introduction

Antidepressant placebo effects — averaging 31–45%, compared to ∼50% response rates to conventional antidepressant medication — pose significant challenges for drug development (1, 2), a process progressively more time-consuming (currently 13 years on average) and expensive ($800 million to $3 billion per new agent) compared to medications for non-central nervous system (CNS) indications (3). Despite innovative clinical trial designs (4) and statistical methods (5, 6) aimed at controlling for this source of noise, the neurobiological mechanisms underlying antidepressant placebo effects are unknown. However, growing evidence suggests that placebos are not just control conditions in clinical trials and that expectations and learning mechanisms associated with their administration activate neurobiological substrates to produce physiological and clinical changes (7). 

Functional neuroimaging studies, stemming primarily from the area of placebo analgesia, have rapidly advanced our knowledge of the mechanisms underlying placebo effects in pain using sophisticated experimental approaches (8). However, similar progress has not yet taken place in the field of psychiatry. In depression, the delayed mechanism of action of antidepressants (9) makes it hard to induce expectancies of fast-acting antidepressant effects. Furthermore, changes in mood states have long temporal dynamics (10), compared to brief and reliable pain manipulations. For these reasons, most experimental studies of antidepressant placebo effects have taken place in the context of antidepressant clinical trials, far from laboratory settings. Some of these difficulties may explain the scarcity of scientific evidence that followed the first neuroimaging studies on antidepressant placebo effects in 2002 (11,), compared to hundreds of studies (13–17) that followed the first neuroimaging study on placebo analgesia published the same year (18). This review will cover some of the methodological approaches used within the pain field and describe some of the challenges encountered by the field of antidepressant placebo effects and the potential opportunities that arise from the fields of neuroimaging and computational neuroscience currently used by other disciplines.

## Theories of the Placebo Effects

Classical theories of the placebo effect, informed predominantly by placebo analgesia experiments, posit that placebo responses are explained by expectancy and conditioning mechanisms (19). While the former understands placebo effects as a product of expectations (e.g. “verbal instructions”), the latter understands then as conditioned responses (CR) through the pairing of a neutral stimulus (e.g., the placebo pill) with an unconditioned stimulus (US, e.g., the active drug). More recently, computational theories of placebo analgesia have suggested that placebo effects can be explained by a predictive coding framework, where the brain has a hierarchical, internally generated model of the world that is compared against incoming sensory stimuli (20). According to predictive coding theories, experiencing a sensation like pain results from bottom-up sensory signals as well as top-down expectancies about pain. The mismatch between these bottom-up and top-down signals is used to refine future expectancies in order to better predict future sensory input. This computational framework suggests that expectancies about pain serve as *priors* on experiences, whereas sensory input forms the *likelihood*. Very strong expectancies are represented by *priors* with low variance, which in Bayesian updating means that incoming information (such as sensory signals) has little effect; the opposite is true of weak or uncertain expectancies. Therefore, strong expectancy *priors* about the effect of a placebo will reduce the amount of learning that occurs from experience. In an experimental test of this model, Grahl et al. (21) fit a Bayesian updating model to two groups of participants who received a placebo treatment with expectations of analgesia. In both groups, the thermal pain delivered during putative ‘treatment’ trials was lower than the pain delivered during control trials; however, for one group, lower pain level was always constant, whereas for the other group it was variable. After participants had learned to associate the ‘treatment’ with lower pain, their pain levels were measured during a test phase where equal levels of pain were either paired or not paired with the ‘treatment’ cues. According to theory, participants receiving variable levels of pain while learning about the effects of the placebo analgesia should have a wider prior during the test phase and be more influenced by sensory pain signals — the likelihood. Accordingly, placebo effects correlated positively with the *precision* of *prior* expectations, and this *precision* was mapped onto the periaqueductal gray (PAG) and the rostral ventromedial medulla. This study showed that pain perception results from the integration of expectancies, in the form of *priors*, and sensory information, in the form of *likelihoods*, and the relative variances of these distributions affects placebo learning at behavioral and neural levels.

Alternative computational accounts have been considered. For example, current evidence suggests that placebo effects can be explained by models of reinforcement learning (RL). These models, and in particular variants of the Rescorla-Wagner model (22), propose that individuals update their expectancies as new sensory evidence is accumulated (e.g. pain), by incorporating a *prediction error* (PE), which signals the mismatch between what it is expected (*expected value*) and what it is perceived (*the reward*). This PE is then scaled by the *learning rate*, a parameter controlling the speed of updating of new sensory evidence and added to the expected value of the next experience. In standard RL, expectations not confirmed by experience are extinguished. However, emerging evidence from placebo analgesia experiments suggests that placebo analgesia arises from mechanisms implicated in *self-reinforcing expectancies*, such as *confirmation biases*, where expectancies are selectively reinforced by predictive cues (e.g., the placebo) only when new experience confirms prior expectations, or discount new evidence otherwise (16). Alternatively, others have suggested that persistent expectancies result from *impaired extinction learning* caused by prefrontal downregulation of RPEs (23).

These different theoretical frameworks have been embedded in many experimental designs of placebo analgesia since its early stages, leading to substantial progress in identifying the cognitive, neural and molecular bases of placebo analgesia. While it remains largely unknown whether similar conceptual frameworks can be applied to the formation of placebo responses across disorders, these experimental approaches have the potential to illuminate new insights into our understanding of antidepressant placebo effects.

## Neuroimaging Approaches to Antidepressant Placebo Effects: Learning From the Field of Placebo Analgesia

### Neuroimaging Models of Placebo Analgesia Effects

The very first neuroimaging study of placebo analgesia measured regional cerebral blood flow (rCBF) with positron emission tomography (PET) to compare the effects of the short-acting µ-opioid receptor agonist remifentanil or a placebo under expectations of analgesia. This study revealed increased brain activity in the rostral anterior cingulate cortex (ACC) for both remifentanil and the placebo conditions. Placebo, but not remifentanil, further increased the connectivity between the rostral ACC and the PAG (18). Since then, many neuroimaging studies have followed this original investigation.

Most commonly, neuroimaging experimental designs of placebo analgesia involved verbal instructions of pain relief (“This is a potent analgesic”) along with an inert treatment (e.g., a topical cream), compared to a control condition—the same inert treatment without expectations of pain relief. During an associative learning phase, the placebo is paired with a low-intensity painful stimulus and the control condition is paired with a high-intensity painful stimulus. Finally, during the test phase — usually conducted during a functional MRI scanning session — both the control and the placebo conditions are paired with a painful stimulus of the same intensity. Under these circumstances, experimenters can test whether pain reports and brain responses are modulated by the patient’s beliefs about the treatment (8). Alternatively, pharmacological conditioning designs have involved the pairing of the relevant stimuli (e.g. pain stimuli, emotionally balanced pictures) and an acute active treatment (e.g. analgesic), during the associative learning phase.(24). While many alternative designs have been used to investigate placebo effects in the context of clinical trials (e.g. *parallel group designs* or *open versus hidden drug design),* this trial-by-trial manipulation of expectancies and sensory inputs (e.g. pain, mood) has been an essential feature of experimental neuroimaging models of placebo analgesia, which has allowed a rapid understanding of the behavioral, neural, molecular, and computational bases of placebo analgesia. 

These studies have demonstrated placebo-induced activation in several cortical areas, such as the ACC and the dorsolateral prefrontal cortex (dlPFC) (18, 25), as well as the descending pain modulating system, involving the hypothalamus, the PAG, and the rostroventromedial medulla, reaching down to the spinal cord (13). More specifically, meta-analytic results have described both placebo-induced reductions in brain responses during painful stimulation in dorsal ACC, insula, thalamus, amygdala, striatum, and lateral prefrontal cortex, as well as placebo-induced increases in activation prior to and during noxious stimulation in the dlPFC and ventromedial PFC, rostral ACC, the midbrain surrounding the PAG, left anterior insula, and the striatum (8). Furthermore, studies using opioid antagonist blockade (26–29) and *in vivo* receptor binding of μ-opioid receptors (30, 31) have extensively confirmed the role of µ-opioid neurotransmission in placebo analgesia (32), and more recently antidepressant placebo effects (33), consistently with the role of the opioid system in pain (34) and mood processing (35). Nowadays, Neurosynth (36) and other related large-scale neuroimaging databases also offer the opportunity to perform comprehensive reverse inference analyses to define the neural correlates of placebo effects. Consistent with the results reported above, when the term “placebo” is entered as a term into a Neurosynth uniformity test, results from 332 studies reveal increased activity present in the dlPFC, dorsal, rostral, and subgenual ACC, the thalamus and the VS.

### Neuroimaging Models of Antidepressant Placebo Effects

**Figure 1:**
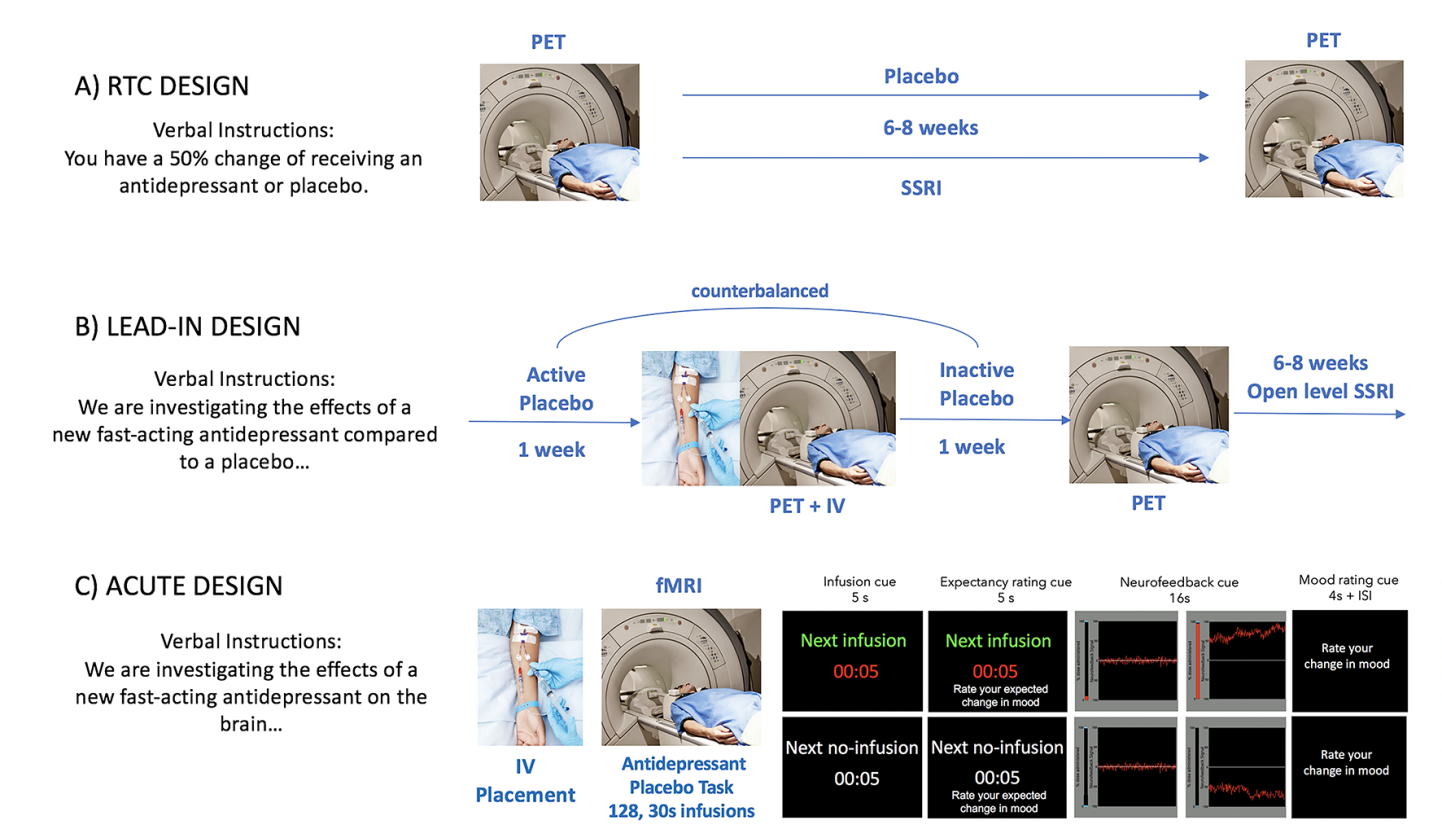
Experimental models of antidepressant placebo effects. Most neuroimaging studies of antidepressant placebo effects fall into one of the following categories: An RCT of active antidepressant vs. placebo, a placebo lead-in phase or an acute placebo manipulation.

The experimental manipulation of expectations of mood improvement as well as its conditioning posits significant challenges. For example, the delayed action of conventional antidepressants limits the possibility of manipulating expectancies acutely. Furthermore, mood, unlike pain—which reliably emerges in response to specific stimuli—is a latent state with complex internal dynamics. For these reasons, most neuroimaging studies have used placebo-induced neuroimaging changes in the context of randomized clinical trials (RCTs) (11,) (pre- and post- placebo mood changes). Although these studies have informed about the biological substrates that underlie antidepressant placebo effects, they have yet to describe a mechanism through which antidepressant expectancies evolve to induce persistent mood changes, like those observed in RCTs. Critical to this aim is the development of novel trial-by-trial manipulations of antidepressant placebo effects. We have recently developed the first paradigm involving a trial-by-trial manipulation of antidepressant placebo effects (37). Here, we will argue that this kind of experimental manipulation is a necessary first step to develop an understanding of placebo effects that is embedded in a conceptual understanding of this phenomenon (**Figure 1**).

#### Parallel Group Designs of Antidepressant Placebo Effects

In the very first study that examined the neural correlates of antidepressant placebo effects, Leuchter et al. (12), used quantitative electroencephalography (QEEG) to compare changes in brain function during a 9-week RCT of fluoxetine or venlafaxine. QEEG data was collected at baseline, after a 1-week placebo lead-in phase, and at 2, 4, and 8 weeks after the start of double- blind treatment. This study showed that by week 2, placebo responders, compared to drug responders, showed increases in prefrontal cordance that significantly diverged from baseline by week 8. Contrary, at week 2, only drug responders showed a significant decrease in prefrontal cordance, which resolved at weeks 4 and 8. This was the first study to demonstrate that despite achieving similar symptomatic improvement, placebo and antidepressant treatments engaged prefrontal function through opposite mechanisms of action, specially at early stages during the course of treatment.

Soon after, Mayberg and colleagues examined the neural correlates of antidepressant placebo effects using fluorodeoxyglucose (FDG) and PET before and after 1 and 6 weeks of fluoxetine or placebo (11). This study revealed that, after 6 weeks of treatment, placebo responders had regional metabolic increases in the prefrontal cortex, ACC, premotor and parietal cortex, posterior insula, and posterior cingulate and metabolic decreases in the subgenual ACC, parahippocampus, and thalamus, whereas drug responders had additional metabolic increases in the brainstem, striatum, anterior insula, and hippocampus (11). 

These two studies represented a major step forward in the investigation of antidepressant placebo effects. Interestingly, and despite using very different neuroimaging modalities with different temporal and spatial resolution, both studies found overall increases in prefrontal activity in response to the drug or the placebo treatments. 

#### Placebo Lead-In Designs of Antidepressant Placebo Effects

In the context of RCTs, parallel group designs often assess symptom stability using a placebo lead-in phase. During this phase, subjects who meet initial screening criteria, but exhibit a 20–25% reduction in symptoms, are usually excluded from participation in the post-randomization phase of the trial (38).

Biomarker studies have used placebo lead-in designs to examine the relationship between neural changes during the placebo lead-in period and the endpoint clinical outcome. An example of this kind of experimental design is the one published by Hunter et al., where they examined the neural responses during a placebo lead-in phase (39). In this case, they found that decreased prefrontal cordance during the placebo lead-in period predicted lower depression severity by the end of the trial in patients assigned to medication. 

More recently, we conducted a study that involved a two-week single-blinded, crossover, randomized placebo lead-in of 2 identical oral placebos (described as having either ‘active’ or ‘inactive’ fast-acting antidepressant-like effects) followed by a 10-week open-label antidepressant treatment (33). In this study, 35 medication-free patients were studied with PET and the µ-opioid receptor-selective radiotracer [^11^C] carfentanil after the ‘active’ and an ‘inactive’ oral placebo treatment. In addition, during the PET scanning session, but only after the active placebo condition, participants were administered 1 mL of isotonic saline intravenously, with instructions of fast-acting antidepressant effects. This study had several interesting findings. First, higher baseline opioid receptor binding in the nucleus accumbens (NAc) was associated with a better treatment response during the 10-week open label antidepressant treatment. Second, clinical responses to the ‘active’ placebo treatment, compared to the ‘inactive’, were associated with increased placebo-induced μ-opioid neurotransmission in the subgenual ACC, NAc, midline thalamus and amygdala. Finally, we found that placebo-induced opioid neurotransmission was associated with better antidepressant treatment response, predicting 43% of the variance in symptom improvement at the end of the antidepressant trial (33). 

**Figure 2:**
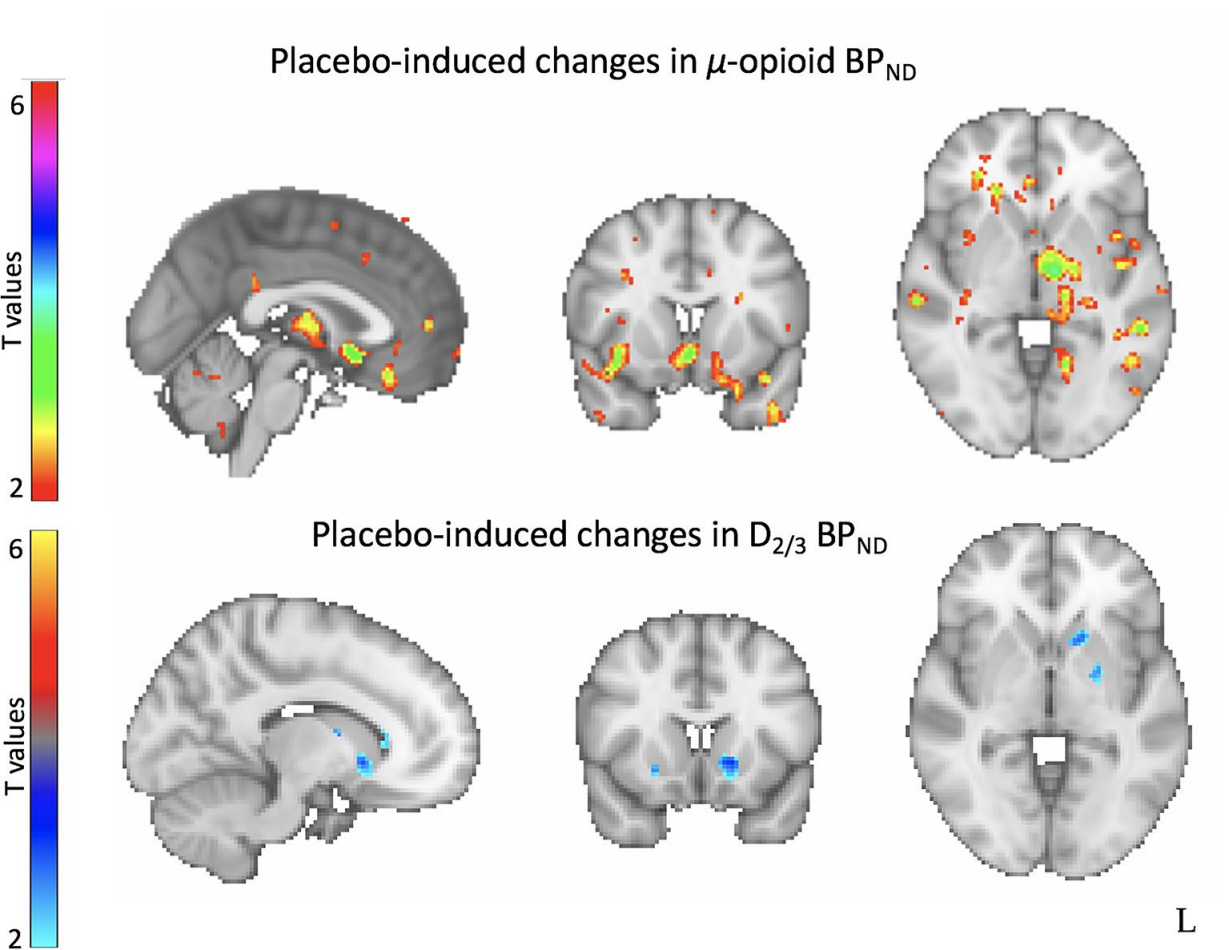
Neural correlates of antidepressant placebo effects. Neuroimaging studies of antidepressant placebo effects using positron emission tomography (PET) have demonstrated increased placebo-induced µ-opioid neurotransmission in the subgenual anterior cingulate cortex (sgACC), the amygdala, the thalamus and the ventral striatum (top) and increased dopamine-induced neurotransmission in the ventral striatum.

In addition, twenty-six patients from the sample described above completed a PET scan with the D_2/3_ receptor-selective radiotracer [^11^C] raclopride after each 1-week inactive and active oral placebo treatment. Here, we found that, compared to a matching sample of healthy controls, patients with depression showed greater D_2/3_ receptor availability in the bilateral ventral pallidum/NAc, and the right ventral caudate and putamen. D_2/3_ receptor availability in the ventral striatum correlated positively with high anxiety (caudal portion) and negatively with anhedonia (rostral portion). Furthermore, we observed increased placebo-induced DA neurotransmission in the ventral striatum. However, these changes were not correlated with the patient’s levels of expectations of improvement or their mood improvement after the I.V. or the oral placebo nor the treatment with 10 weeks of antidepressants (40) (**Figure 2**). These results suggested that antidepressant placebo effects resulted in increased opioid and DA neurotransmission in regions involved in emotional and reward processing, mostly subcortically. However, as suggested by prominent reward theories (41), while both neurotransmitter systems are released in response to the administration of placebos, the mesolimbic dopamine system may be involved in the placebo ‘wanting’ — or the incentive salience that motivates approach — while the μ-opioid system may be involved in the placebo ‘liking’ — the physiological response to a hedonic stimuli.

The same patients also completed a resting state functional connectivity (RSFC) after each of two different ‘active’ and ‘inactive’ placebos (42). In this case, we found that increased RSFC in the rostral ACC within the salience network predicted both better response to the active, compared to the inactive placebo, and to the 10-week antidepressant treatment. Furthermore, using machine learning we showed that increased RSFC in the rostral ACC significantly predicted individual responses to placebo administration. These results suggested that increased RSFC in the rostral ACC, the most reliable marker of treatment response in depression across multiple treatments (43), as well as placebo analgesia (18, 24), seems to play a significant role in the formation of antidepressant placebo effects.

#### Trial-By-Trial Designs of Antidepressant Placebo Effects

We recently developed a new Sham Neurofeedback fMRI Task (37). This task features a within-subject trial-by-trial manipulation of two putative components of the placebo antidepressant effect: the expectancy of mood improvement and its reinforcement. During the expectancy manipulation, patients were presented with a drug infusion or no-infusion cue, which instructs patients about the imminent infusion of the “fast-acting antidepressant” (intravenous saline) or its absence, respectively. During the reinforcement manipulation, patients were presented with the display of sham neurofeedback signal of positive or negative valance during 20 s, with instructions that it reflected changes in brain activity in response to the drug infusions. Patients were asked to rate their expectations of mood improvement and their actual mood improvement after each expectancy and reinforcement manipulation, respectively, using a 7-point Likert scale (**Figure 1**). 

Results from this study in 20 patients with MDD demonstrated the feasibility of manipulating fast-acting antidepressant effects. As expected, patients reported higher expectancy ratings during the placebo infusion condition (expecting a drug infusion as opposed to no infusion), and higher mood ratings during the drug infusion cue, compared to the no-infusion cue, and following the display of positive sham neurofeedback, compared to negative. Furthermore, the positive effect of neurofeedback on reported mood was enhanced when expectancies were high, as reflected in a positive two-way interaction. 

The presentation of neurofeedback of greater magnitude recruited greater blood-oxygen-level dependent (BOLD) responses in the bilateral ventrolateral and dorsolateral PFC. Furthermore, greater increases in β-endorphin plasma levels during the task were associated with higher expectancy ratings during the placebo condition, compared to the no-infusion condition, and higher mood ratings during positive neurofeedback, compared to negative.

In our opinion, this trial-by-trial manipulation is an essential first step to decoding the neural representation of antidepressant placebo effects, by dissecting the different components of the placebo response and aiding the development of computational models which might provide new opportunities to disambiguate this complex phenomenon. For example, expectancy ratings during the Sham Neurofeedback fMRI Task could be fit to models of RL where learned expected values for each trial type are updated every time the “antidepressant” infusion cue is presented and an outcome (positive or negative neurofeedback) is observed. This updating is based on the following equation: *Q*_t + 1_(*s*) = *Q*_t + 1_(*s*) + αδ*_t_*, where *Q* *_t_* (*s*) is the learned expected value of improvement at trial *t*, α is a learning rate, and δ is the difference between the actual and expected outcome (RPE): δ*_t_* = *r* *_t_* – *Q* *_t_*(*s*), where, *r* *_t_* is the actual reward outcome (positive vs. negative neurofeedback). These values are used to make choices (such as ratings of expectation of improvement) according to a sigmoid choice rule with two free parameters: β (*stochasticity*) and K (*choice bias*). The estimation of such parameters and derived values (e.g., expected values, RPE, etc.) — which cannot be accessed with descriptive approaches alone — can then be mapped onto the neural response during the Sham Neurofeedback fMRI Task. This trial-by-trial information is likely to provide new opportunities to disambiguate placebo responses. Furthermore, this transdiagnostic RL framework may apply to other clinical conditions where placebo effects are also prevalent, notably anxiety disorders, Parkinson’s Disease, and various forms of persistent pain, but also schizophrenia, substance use disorders and surgeries (44, 45).

## Computational Approaches to Antidepressant Placebo Effects

**Figure 3:**
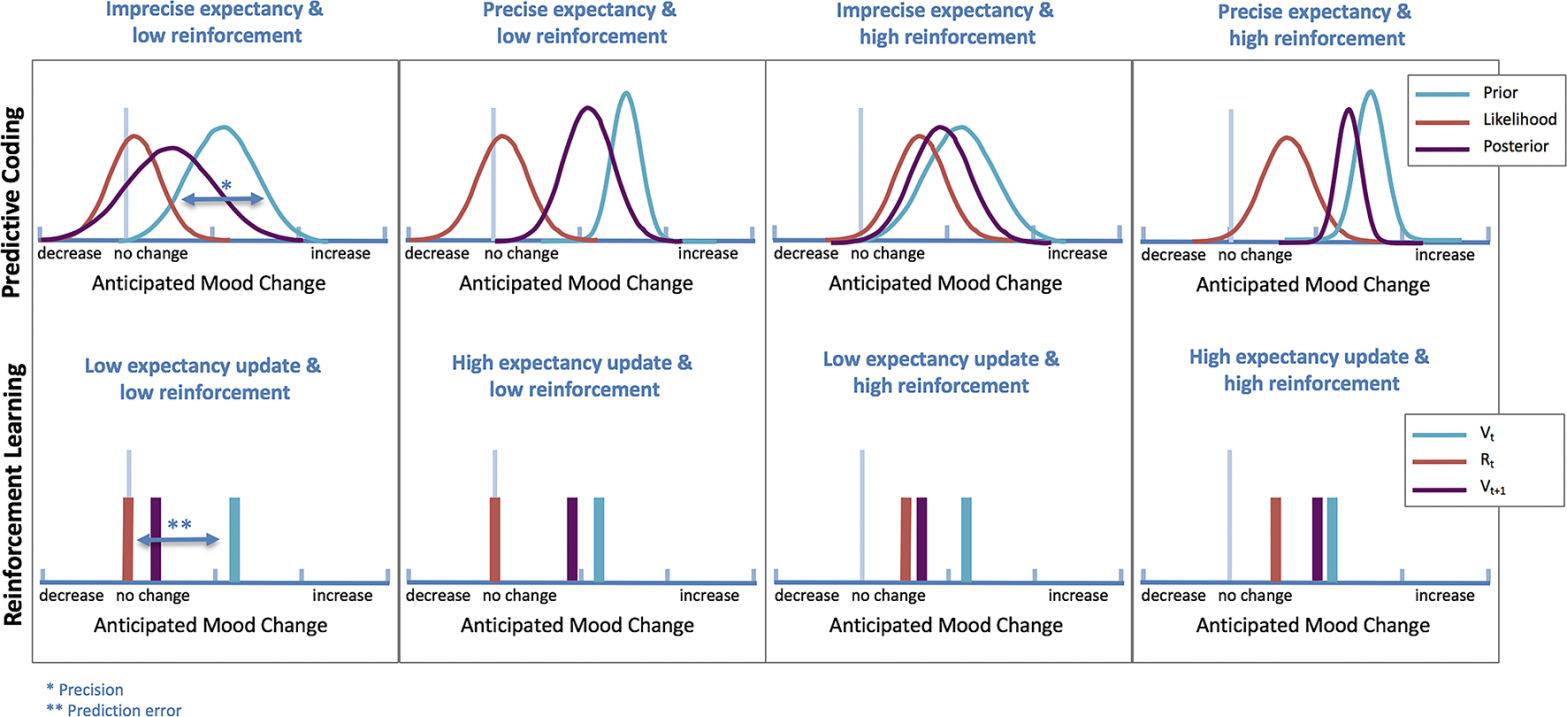
Computational theories of antidepressant placebo effects. Illustration of differences in antidepressant placebo effects under predictive coding (top) and RL (bottom) theories (simulated data). In predictive coding theories, expectancy updating based on the strength of expectancy of improvement is operationalized as differences in precision (narrowness of prior), while in RL, is operationalized by incorporating a prediction error (PE), which signals the mismatch between what it is expected (Vt) and what it is perceived (Rt).

Whereas computational theories have not yet been applied to models of antidepressant placebo effects, recent evidence supports a relationship between RL and mood, which opens the possibility that antidepressant placebo effects might indeed result from RL mechanisms (46, 47). Expectations and PEs have shown to affect self-reported mood on a trial-to-trial basis (48), and mood can bias how people perceive and learn from rewards (46, 47). This bi-directional relationship between learning and mood is likely to play a significant role in the formation of antidepressant placebo effects (**Figure 3**). 

RL models of antidepressant placebo effects are therefore likely to be influenced by features frequently affected in patients with depression. For example, patients with depression may show a reduction in the *primary sensitivity* to rewards (reduced consummatory anhedonia) and/or alterations in their ability to *learn* from positive or reward feedback. Furthermore, patients with depression might show exaggerated processing of negative or aversive feedback (49). These alterations in the processing of positive and negative feedback in patients with depression could also have implications for nocebo effects in this disorder as well. Therefore, RL models of antidepressant placebo effects might need to incorporate additional features such as *reduced sensitivity to positive feedback and differential sensitivity to positive versus negative feedback*. Models that account for these biases can adjust outcome processing or learning based on the valence of outcomes (by modulating learning rates or sensitivities to outcomes for positive and/or negative feedback) or prediction errors (by estimating separate learning rates for positive versus negative prediction errors).

Finally, improved mood may increase processing and learning from positive outcomes, biasing learning towards more positive learning with initial improvements in mood (46). Therefore, models of antidepressant placebo effects may also benefit from including *bidirectional influences between mood and learning*. This kind of biases create a feedback loop where initial improvements in mood, through biasing learning in positive direction, lead to more positive future mood states, providing a potential mechanism for the perpetuation of placebo responses.

## Conclusion

This review has identified several challenges and opportunities that have emerged from early research investigating the neurobiology of antidepressant placebo effects and new computational approaches. As discussed, much can be learned from experimental approaches extensively used by other disciples.

In the future, the formalization of computational models of antidepressant placebo effects and other psychiatric conditions may provide a fruitful approach to map learning-based models of antidepressant placebo effects onto the underlying neural mechanism. The delineation of such a computational framework and associated neural circuits and neurotransmitters systems will open new *translational opportunities* to promote treatment response by stimulating placebo-related networks as new targets for mood improvement. From the perspective of drug and therapy development, *inhibiting placebo responses* could help separate drug-specific and “non-specific” treatment effects. Higher signal and less noise in RCTs would, therefore, result in substantial savings by reducing the samples sizes necessary to achieve significant differences between active and inactive treatments. As discussed, a first step towards this aim is the use of model-based experimental approaches that disentangle the different elements involved in this complex phenomenon, including those shared by other disorders and those that are mood specific. 

Furthermore, the development of software tools and platforms that might provide access to high quality clinical multi-disciplinary data may allow the development of computational brain models useful in clinical practice (for example, Virtual Brain: https://www.thevirtualbrain.org/tvb/zwei ). Such neurocomputational models could potentially be used to help identify key subject-specific mechanisms of placebo responses that might impact treatment response broadly. This approach is more likely to account for individual differences in placebo responses, a phenomenon that is subject to both intra-individual and inter-individual variability. Consistently, recent evidence suggests that functional organization within individual subjects is idiosyncratic and relatively robust to changes in brain state and provides meaningful information beyond group averages (50–52). This progress in key to the development of biomarkers of treatment and personalized medicine.

## Author Contributions

VB and MP contributed to writing, reviewing and making figures and tables for this review article.

## Funding

This work was supported by a K23 MH108674 (MP) and a T32 MH019986 (VB).

## Conflict of Interest Statement

The authors declare that the research was conducted in the absence of any commercial or financial relationships that could be construed as a potential conflict of interest.
